# Comparison of Primary Care Experiences in Village Clinics with Different Ownership Models in Guangdong Province, China

**DOI:** 10.1371/journal.pone.0169241

**Published:** 2017-01-05

**Authors:** Shanshan Feng, Leiyu Shi, Jiazhi Zeng, Wen Chen, Li Ling

**Affiliations:** 1 Faculty of Medical Statistics and Epidemiology, School of Public Health, Sun Yat-sen University, Guangzhou, China; 2 Sun yat-sen Center for Migrant Health Policy, Sun Yat-sen University, Guangzhou, China; 3 Faculty of Health Management, School of Health management, Guangzhou Medical University, China; 4 Department of Health Policy and Management, Bloomberg School of Public Health, Johns Hopkins University, Baltimore, United States of America; University of Ottawa, CANADA

## Abstract

**Objectives:**

In order to improve the quality of services at village clinics (VCs), which are important primary care service providers in rural China, the Chinese government has encouraged the township hospitals to own and manage VCs. There are currently three models of ownership and management of VCs: township hospital-owned and -managed (HVC), village committee-owned and -managed (VVC), and private-owned and -managed (PVC). This study aims to examine the association between these ownership models of VCs and patients' primary care experiences.

**Methods:**

Villagers were selected by multistage stratified sampling and their experiences with primary care were measured using the Primary Care Assessment Tool—Adult Edition (PCAT-AS). Data were collected through face-to-face interviews and the questionnaires administered by investigators in the cross-sectional study from February to April 2015. The PCAT scores were compared among the three models by covariance analysis, and multiple linear regression was used to analyze factors associated with the PCAT total scores.

**Results:**

A total of 1491 questionnaires were collected. After controlling for covariates, HVCs reported the highest PCAT scores and satisfaction rate. In terms of the domains, HVC reported the highest scores in the coordination and comprehensiveness domains, while PVC had the highest scores in the first contact-accessibility domain. Multivariate linear regression showed that HVC, married participants, aged 60 and older, satisfied with the services, receiving six or more visits, and those with medical expenditures over 20% of their total family expenditures, were also positively associated with better primary care quality.

**Conclusions:**

This study demonstrates that villagers receiving medical care at HVCs perceived better primary care than those at PVCs and VVCs. In order to improve the quality of primary care at VCs, it is necessary to increase government subsidies for public service packages, tighten the township hospital's supervision of PVCs and VVCs, and develop performance-based incentive plans to motivate improvements in the accessibility of HVCs.

## Introduction

In many countries, research on health care reform has proven that a strong primary care system forms a solid foundation to provide accessible and affordable primary care to residents and improves the performance of the entire health system [1,2]. China’s 2009 health care reform aims to achieve the objective of “health for all” by expanding basic health insurance coverage and strengthening the primary care system. The improvement of the health care system in rural areas was regarded as the core of the reform plan [3]. Three tiers of health providers in rural areas, including county hospitals, township hospitals, and village clinics (VCs), offer health services for rural residents. VCs act as the frontline providers for the villagers and are designed to provide accessible, continuous, and comprehensive basic health services to protect villagers’ health [3,4]. As a result of implementing a series of initiatives to improve the service capacities of VCs, the number of outpatients visits in VCs reached over two billion, 27.4% of all health care visits nationally in 2013 [5,6].

In rural China, all township hospitals are fully funded by the government. From the 1960s to 1970s, the VCs were organized on the basis of community economies and run by village committees. However, after the reform of the economic system in the 1980s, the people's communes were dissolved and the original economic foundation for VCs was gone. Gradually, some VCs became privately owned [7]. Consequently, there were two major village clinic models: village committee-owned and -managed village clinics (VVC) and private-owned and -managed village clinics (PVC). Since 2010, the Chinese government has been promoting the integration of health services, and thus encouraged township hospitals to own and manage the VCs (HVC) in order to strengthen the supervision of VCs and improve the quality of service they offered [8]. Currently, three major models are in place: VVC, PVC, and HVC [4]. All the VCs are run under the HVC model in the townships that provide integrated primary care. In the rest of the townships, VCs follow the VVC or PVC model. In 2013, 9.2% of VCs were HVCs, 66.3% were VVCs, and 24.5% were PVCs in China [6]. Whether HVCs improve the quality of the VCs is the key focus area of this research.

Though the services and models of primary care in different countries are substantially influenced by national context and culture, internationally, a consensus has been achieved on the function of primary care. The National Institute of Health (NIH), the World Health Organization (WHO), and many experts have defined the characteristics of primary care as accessibility, first-contact, comprehensiveness, continuity, and coordination [9–12]. It has also been widely accepted by experts that the participants' self-reported perspective is a reliable means to assess the quality of primary care [13–16].

Previous primary care research has focused on urban primary care providers, while few studies were carried out to evaluate the quality of care among rural providers [17–21]. The unique circumstances in rural areas as well as the fact that rural population accounts for two thirds of the total population in China indicate the importance of research on primary care in rural areas. Moreover, such research is critical to improve the quality of primary care services and promote the health of rural residents. Previous research has explored the influence of institution types, institution characteristics, and doctor’s professionalism on the quality of primary care services [19–23] but rarely focused on the influence of ownership and management models, particularly in rural China [18,24]. This study is the first to examine the association between ownership models of rural grassroots medical institutions and participants' primary care experiences. The study area Guangdong province, is a microcosm of China to some extent in terms of economic development, where the combination of the developed Pearl River delta region and less developed surrounding areas is similar to the economic distribution of the whole China.

## Methods

### Survey design and procedures

**Fig 1:**
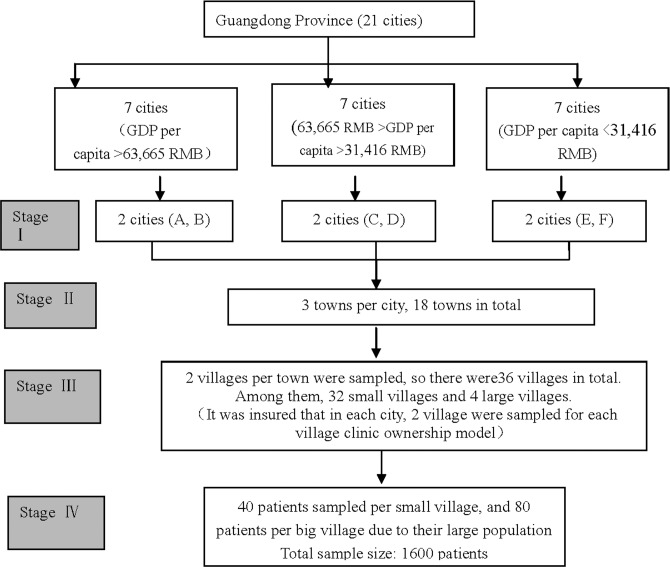
The Flow Chart of Sampling. Three inclusion criteria were established in the selection of study participants: 1. The study participants should be aged 18 or older. 2. The study participants must sign the written consent. 3. The study participants must have visited a VC at least once in the past year. The interviewers were postgraduates and undergraduate students from the School of Health Management of Guangzhou Medical University. They were trained on how to conduct the survey in order to improve the completeness and consistency of the investigation. The interviewers were introduced to the participants by local acquaintances, and they could be village heads, women's directors, or the respected senior citizens. The data were collected through face-to-face interviews, and the questionnaires were administrated by the investigators at respondents’ home. It took about 20 minutes to finish the survey, and a gift was given to each participant as a token of appreciation for the participation.

This cross-sectional study was conducted in rural of Guangdong Province, China from February to April 2015. Multistage stratified sampling was adopted for this study (Fig 1). In the first stage, 21 cities in Guangdong province were divided into three groups according to their geographic location and economic development. Two cities were randomly chosen from each group, with six cities ultimately included. In the second stage, three towns were chosen from each of the six cities, including a township that offers integrated primary care for its residents. In the third stage, two villages were chosen from each of the 18 towns. That is, three towns and six villages were sampled from each city. Among the six villages, two villages run the village clinic in the VVC model, two in the HVC model, and two in the PVC model. Among them, there are 32 small villages and four large villages (one village in the PVC model and three villages in the VVC model). Because there were four large villages that had twice the population of the other villages, two village clinics were chosen in the large villages. In the fourth stage, 40 study participants from each average-sized village and 80 from each large village were selected. Therefore, a total of 1600 study participants were identified in this study. This sample size was determined based on findings from existing published literature. Current research shows that for such an analysis, a minimum sample size of 300 per group is needed for a significance level of 5% with a power of 90%. In this study, the sample size for each model was above 432, which was regarded adequate to provide good statistical power [17–18, 25].

HVCs are publicly funded and regarded as an affiliated sector of a township hospital. The administrative hospitals pay the salaries of the village doctors, supervise and guide their work, and manage the VCs’ profits [7]. As for PVCs, village doctors own and manage the clinics independently, and take the sole responsibility for profits and losses. The government provides subsidies to village doctors for purchasing basic public health services, including chronic disease management, maternal and child care, geriatric care, health records, health education, communicable disease prevention and reporting, immunization, and mental health care [26], and the subsidies are allocated based on providers’ performance. However, government subsidies only make up a very small proportion, and the major income of a PVC comes from medical services provision. Township hospitals and the village committees play very minor roles in supervision and stewardship of the PVCs [27]. As for VVCs, they are owned and managed by the village committees, which are self-administrative organizations at the village level in China. The VVC is solely responsible for profits and losses at the VC [7,27,28]. A summary of these three models is presented in Table 1.

**Table 1:** Models of organization and management of village clinics.

	HVC	VVC	PVC
**Ownership**	Township hospital	Village committee	Individual village doctors
**Legal representation**	Dean of township hospital	Director of village committee	Individual villager
**Manpower employed by**	Employed by township hospital	Employed by village committee	Self-employment
**Service provision**	Basic medical services and public health services	Basic medical services and public health services	Basic medical services and public health services
**Supervised by**	Township hospitals	Village committee	Absence of supervision
**Salary structure**	Basic salary plus performance related incentives	Government subsidy plus medical service provision income	Government subsidy plus medical service provision income
**Income distribution**	Turns over to township hospitals	Takes sole responsibility of profits and losses	Takes sole responsibility of profits and losses

Note: HVC = township hospital-owned and -managed village clinic; VVC = village committee-owned and -managed village clinic; PVC = private-owned and -managed village clinic.

### Research instrument

The questionnaire used in this research was the Primary Care Assessment Tool-Adult Edition (PCAT-AS), which was designed by Barbara Starfield and Leiyu Shi at the Primary Care Policy Center at Johns Hopkins University. This tool has been widely used and tested in a number of countries and regions such as the US, Canada, Japan, Taiwan, South Korea, and China [17,20–25, 29–30]. The Chinese versions of the PCAT questionnaire was tested and proved to have good reliability and validity [25,31]. The research team obtained the author’s consent to use the questionnaire.

PCAT-AS was designed to be consistent with the core functions of primary care. Thirty-six items were developed to assess the seven domains of study participants' primary care experiences, of which there were four core domains (with two sub-domains assessing structure and content): First Contact (accessibility and utilization), Continuity, Coordination (information and referral systems), and Comprehensiveness (service availability and service provided), and three scales for Community Orientation, Family Contentedness, and Culturally Competency. A Four-point Likert-type scale was adopted as the measurement scale where 1 = definitely not, 2 = probably not, 3 = probably, 4 = definitely, 9 = not sure/don't know. The score of each domain is the sum of scores of each item within the domain. The items coded 9 were assigned value 2 except for comprehensiveness (services provided) section where it was assigned to 0. The PCAT total score is the sum of scores obtained in seven domains. Higher scores indicate better patient primary care experience, according to the PCAT Manual [32,25].

In addition, the questionnaire included socio-demographic characteristics such as age, gender, marital status, education, occupation, and self-evaluated economic status. Items measuring health service utilization were included as well, such as satisfaction with providers, self-evaluated health status, number of VC visits in the past year, proportion of medical expenditures to total family expenditures, and health insurance status. The medical expenditures refer to costs spent in all health facilities.

### Data analysis

Data were double recorded by the software Epidata 3.1, and SPSS 18.0 was used to conduct statistical. We conducted Chi-square tests to compare the socio-demographic characteristics and health care utilization of study participants among the three types of VCs. Analysis of covariance was employed to compare the adjusted PCAT domain scores and total scores among the three VC models. We included the socio-demographic and health service utilization variables that were statistically significant as the covariates of the model. Pair-wise comparison was performed to test the scores of three models with LSD-t (Least Significant Difference) test. Then, multivariate linear regression was performed to explore the relationship between types of VCs and the perceived primary care quality (represented by the PCAT total scores), controlling for respondents’ personal and health care characteristics (i.e. age, gender, marital status, education, occupation, self-evaluated health status, self-evaluated economic status, number of health care visits in the past year, proportion of medical expenditures to total family expenditures, and satisfaction with the health care experience). Binary logistic regression was used to evaluate the relationship between types of VCs and satisfaction with the health care experience, controlling for PCAT total scores and other personal characteristics. We used the ‘Enter Method’ to include variables. In the analyses, P<0.05 was considered to be statistically significant.

### Ethical consideration

Ethical approval was obtained from the Ethics Committees of the Guangzhou Medical University. In this face-to-face survey study, all study participants were informed of the purpose of the study and had the right to leave the interview at any time. All study participants were required to give written consent before the interview.

## Results

A total of 1600 study participants were interviewed and 1491 (93.2%) qualified questionnaires were collected for the final analysis. We analyzed data from 477 study participants in the PVC, 582 study participants in the VVC, and 432 study participants in the HVC. The socio-demographic characteristics and health care utilization of sampled study participants in VVCs, HVCs, and PVCs are reported in Table 2. No significant difference was observed in measures of gender, marital status, education, occupation, self-evaluated health status, or health insurance among participants in the three groups, but significant differences were reported on the following five measures: age, self-evaluated economic status, number of VC visits in the past year, satisfaction, and proportion of medical expenditures to total family expenditures. Nearly all respondents in rural Guangdong Province were insured (98.8%).

**Table 2:** The comparison of demographic characteristics and service utilization indicators among three types of VCs.

Variables			N(%)	PVC(%)	VVC(%)	HVC(%)	*χ*^*2*^	P
			n = 1491	n = 477	n = 582	n = 432		
Socio-demographic characteristics								
Gender	Male		697(46.7)	213(44.7)	269(46.2)	215(49.8)	2.489	.288
	Female		794(53.2)	264(55.3)	313(53.8)	217(50.2)		
Age	18–34		591(39.6)	207(43.4)	214(36.8)	170(39.4)	21.003	.000
	35–59		644(43.2)	216(45.3)	260(44.7)	168(38.9)		
	≥60		256(17.2)	54(11.3)	108(18.6)	94(21.8)		
Marital status	Married		1024(68.7)	311(65.2)	403(69.2)	310(71.8)	4.677	.096
	Unmarried (single/divorced/widowed)		467(31.3)	166(34.8)	179(30.8)	122(28.2)		
Education	Junior high school and lower		884(59.3)	291(61.0)	347(59.6)	246(56.9)	1.593	.451
	Senior high school and higher		607(40.7)	186(39.0)	235(40.4)	186(43.1)		
Occupation	Agricultural employee		544(36.5)	185(38.8)	218(37.5)	141(32.6)	4.083	.130
	Non-agricultural employee		947(63.5)	292(61.2)	364(62.5)	291(67.4)		
Self-evaluated economic status	Good		212(14.2)	61(12.8)	79(13.6)	72(16.7)	11.260	.024
	Medium		1057(70.9)	351(73.6)	426(73.2)	280(64.8)		
	Low		222(14.9)	65(13.6)	77(13.2)	80(18.5)		
Health service utilization								
Self-evaluated health status	Good		742(49.8)	229(48.0)	311(53.4)	202(46.8)	9.028	.060
	Fair		618(41.4)	214(44.9)	218(37.5)	186(43.1)		
	Bad		131(8.8)	34(7.1)	53(9.1)	44(10.2)		
Number of VC visits	≤6		1168(78.3)	378(79.2)	484(83.2)	306(70.8)	22.548	< .001
	>6		323(21.7)	99(20.8)	98(16.8)	126(29.2)		
Satisfaction	Satisfied		1006(67.5)	283(59.3)	401(68.9)	322(74.5)	24.777	< .001
	Dissatisfied		485(32.5)	194(40.7)	181(31.1)	110(25.5)		
Health insurance	Yes		1473(98.8)	471(98.7)	574(98.6)	428(99.1)	0.434	.805
	No		18(1.2)	6(1.3)	8(1.4)	4(0.9)		
Proportion of medical expenditures to total family expenditures	<20%		1242(83.3)	368(77.1)	490(84.2)	384(88.9)	23.007	0.000
	≥20%		249(16.7)	109(22.9)	92(15.8)	48(11.1)		

Note: HVC = township hospital-owned and managed village clinic; VVC = village committee-owned and managed village clinic; PVC = private-owned and managed village clinic.

Because the Coordination (referral system) domain could only be answered by study participants who visited large hospitals (54.9% of the study participants), the PCAT Total Score was the sum of all domains except for Coordination (referral system), according to the PCAT Manual. PCAT total scores and individual domain scores are reported in Table 3. Five socio-demographic and health service utilization variables that were statistically significant were included as covariates. Analysis of covariance was used to calculate the adjusted PCAT scores and scores of all domains. As for the quality of primary care, HVCs were given the highest score compared to PVCs and VVCs (90.00, 87.43, and 86.88, respectively), and the distinction was not statistically significant between VVCs and PVCs.

**Table 3:** Primary care quality scores for three types of VCs, adjusted.

Domain	Mean				T		F(all)
	VVC	PVC	HVC	V-P^a^	V-H^b^	P-H^c^	
First contact-utilization	7.73	8.27	8.47	5.888^※※※^	5.909^※※※^	0.162	24.557^※※※^
First contact-accessibility	11.96	12.80	11.42	5.449^※※※^	3.520^※※※^	8.418^※※※^	35.630^※※※^
Continuity	11.35	11.69	10.47	2.285^※※※^	5.84^※※※^	7.69^※※※^	31.304^※※※^
Coordination (Referral system)	10.13	10.41	10.12	1.497	0.047	1.263	1.339
Coordination (Information system)	8.03	8.09	9.02	0.149	6.801^※※※^	6.312^※※※^	27.026^※※※^
Comprehensiveness (Services available)	12.53	13.16	14.01	2.576^※^	6.061^※※※^	3.183^※※^	18.314^※※※^
Comprehensiveness (Services provided)	12.31	11.70	12.45	3.635^※※※^	0.167	.001	7.778^※※※^
Family-contentedness	8.43	8.66	8.58	1.638	1.127	0.477	1.470
Community Orientation	6.11	5.84	7.17	1.679	7.336^※※※^	9.967^※※※^	40.700^※※※^
Cultural Competence	8.44	7.19	8.30	10.802^※※※^	1.769	8.198^※※※^	62.991^※※※^
Total score	86.88	87.43	90.00	0.564	3.095^※※^	2.360^※^	4.815^※※^

Notes: 1. HVC = township hospital-owned and -managed village clinic; VVC = village committee-owned and -managed village clinic; PVC = private-owned and -managed village clinic.

2. Total Scores excludes Coordination (Referral system), because this section was only answered by a fraction of patients who had the experience of visiting large hospitals.

3. a: LSD-t Test comparing VVC with PVC b: LSD-t Test comparing VVC with HVC c: LSD-t Test comparing PVC with HVC.

4. *P <0.05. **P <0.01, ***P<0.001.

HVCs received the highest scores compared to PVCs and VVCs in the domains of Coordination (information system 9.02, 8.09, and 8.03, respectively), Comprehensiveness (14.01, 13.16, and 12.53, respectively), and Community-orientation (7.17, 5.84, and 6.11, respectively). PVCs had the highest scores in the domain of First Contact, especially, First Contact Accessibility. VVCs were between HVCs and VVCs in the domain of First Contact Accessibility and Continuity. The three models were not statistically different in the domains of Coordination (referral system), Family Centeredness, and Cultural Competence.

The multivariate linear regression was performed using the PCAT total score as the dependent variable and 10 independent variables (gender, age, marital status, education, self-assessed economic status, ownership and management model, satisfaction, proportion of medical expenditures to total family expenditures, number of VC visits in the past year, and self-evaluated health status). Indicator variables were set for variables of ownership models. We used the Enter Method to include variables. The ownership and management model was an influencing factor, and HVCs had an effective influence on the total score compared with VVCs and PVCs (β = 2.495, P < .005), and married study participants (β = 2.329, P < .005) tended to have better perceived primary care experience. However, patients satisfied with VCs (β = 6.640, P < .001), those who visited medical facilities with higher frequency (β = 4.636, P < .001), and those with medical expenditures over 20% of total family expenditures (β = 4.507, P < .001) tended to report better primary care experiences. The analysis results, including βs, adjusted βs, and their 95% CIs (confidence intervals), are shown in Table 4.

**Table 4:** Linear regression analysis on primary care assessment total scores.

	Univariate		Multivariate	
Variables	*β* (95% CI)	P	*β* (95% CI)	P
**Ownership and management models**				
VVC (ref)				
PVC	.823(-.782,2.429)	0.314	0.836(-0.698,2.370)	0.285
HVC	3.214(1.563,4.864)	0.000	2.495(0.913,4.077)	0.002
**Socio-demographic characteristics**				
**Age (years)**				
18~59 (ref)				
>60	1.841(0.050,3.631)	0.044	-1.735(-3.603,0.133)	0.069
**Gender**				
Male (ref)				
Female	1.511(0.157,2.864)	0.029	1.249(-0.030,2.528)	0.056
**Marital status**				
Unmarried (single/divorced/widowed, ref)				
Married	3.970(2.526,5.414)	0.000	2.329(0.820,3.839)	0.003
**Level of education**				
Junior high school or below (ref)				
Senior high school or above	-3.359(-4.725,-1.993)	0.000	-1.026(-2.567,0.515)	0.192
**Self-evaluated economic status** Good (ref)				
Fair and bad	1.311(-0.624,3.246)	0.184	1.058(-0.800,2.916)	0.264
**Health-Service utilization**				
**Self-rated health status**				
Good (ref)				
Fair and poor	1.563(0.213,2.913)	0.023	-0.219(-1.596,1.157)	0.755
**Satisfaction** Dissatisfied (ref)				
Satisfied	7.114(5.716,8.511)	0.000	6.640(5.240,8.040)	0.000
**Proportion of medical expenditures to total family expenditures**				
<20% (ref)				
≥20%	4.916(3.091,6.741)	0.000	4.507(2.695,6.319)	0.000
Number of VC visits				
≤6 (ref)				
>6	6.210(4.446,7.974)	0.000	4.636(2.832,6.440)	0.000

Note: HVC = township hospital-owned and -managed village clinic; VVC = village committee-owned and -managed village clinic; PVC = private-owned and -managed village clinic.

Binary logistic regression was performed using satisfaction as a dependent variable, and 10 independent variables (gender, age, marital status, education, self-assessed economic status, ownership and management model, satisfaction, proportion of medical expenditures to total family expenditures, number of VC visits in the past year, and self-evaluated health status). We used the ‘Enter Method’ to include variables. Patients’ satisfaction with HVCs and VVCs was higher than that of PVCs (74.5% vs. 68.9% vs. 59.3%, P < .001). Study participants who gave higher PCAT scores, had less expenditures, better economic conditions, lower education, and were older than 60 tended to report greater satisfaction with their care experience. The analysis results, including ORs (odds ratios), adjusted ORs, and their 95% CIs, are shown in Table 5.

**Table 5:** Binary logistic regression analysis on satisfaction.

	Univariate		Multivariate	
Variables	*OR* (95% CI)	P	*OR* (95% CI)	P
**Ownership and management models**				
VVC (ref)				
PVC	0.658(0.511,0.848)	0.001	0.669(0.511,0.878)	0.004
HVC	1.321(1.000,1.746)	0.050	1.167(0.864,1.576)	0.314
**Socio-demographic characteristics**				
**Age (years)**				
18~59 (ref)				
>60	2.595(1.843,3.654)	0.000	2.287(-1.552,3.372)	0.000
**Gender**				
Male (ref)				
Female	1.155(0.929,1.436)	0.194	1.091(0.864,1.378)	0.465
**Marital status**				
Unmarried (single/divorced/widowed, ref)				
Married	1.499(1.192,1.885)	0.001	1.119(0.857,1.463)	0.409
**Level of education**				
Junior high school or below (ref)				
Senior high school or above	0.558(0.448,0.695)	0.000	0.627(0.477,0.824)	0.001
**Self-rated economic status**				
Good (ref)				
Fair and bad	0.599(0.427,0.839)	0.003	0.583(0.406,0.836)	0.003
**Health-Service utilization**				
**Self-rated health status**				
Good (ref)				
Fair and Poor	0.948(0.763,1.178)	0.630	0.813(0.633,1.043)	0.104
**PCAT score**				
1(<79) (ref)				
2(80–87)	2.085(1.559,2.789)	0.000	2.057(1.516,2.793)	0.000
3(88–98)	3.135(2.295,4.283)	0.000	3.164(2.285,4.380)	0.000
4(>99)	3.798(2.752,5.243)	0.000	3.799(2.682,5.380)	0.000
**Proportion of medical expenditures to total family expenditures**				
<20% (ref)				
≥20%	0.708(0.531,0.945)	0.019	0.600(0.435,0.829)	0.002
Number of VC visits				
≤6 (ref)				
>6	1.392(1.031,1.880)	0.031	1.017(0.721,1.435)	0.924

Note: HVC = township hospital-owned and- managed village clinic; VVC = village committee-owned and -managed village clinic; PVC = private-owned and -managed village clinic.

## Discussion

This research is the first to examine the association between the ownership models of rural grassroots medical institutions and participants’ primary care experiences in China using the validated PCAT instrument. The study found that HVCs achieved higher primary care performance than PVCs and VVCs. In previous research, a study from South Korea reported higher scores in private clinics than in cooperated clinics [29].^.^ Harry Wang compared participants' experience in community health centers of three different management and ownership models and found that the government-owned and -managed community health centers received the highest PCAT scores, especially on the domains of Coordination and Acceptability [18]. Therefore, no generalization can be made about the influence of ownership and management model on the quality of primary care in rural areas. At the same time, regional economy, culture, health service system, and health management schemes are all influencing factors.

This research found that participants at HVCs received higher PCAT total scores than those at PVCs and VVCs, especially on Coordination and Comprehensiveness. There are several possible explanations for the high PCAT total scores for HVCs. First, the comprehensive reform of primary care in 2013 encouraged village doctors, general practitioners, public health doctors, and nurses from VCs and township hospitals to build teams and work together. Our research found that the team cooperated better if VCs were under the administration of township hospitals, and consequently residents would receive better services. Meanwhile, health workers got more opportunities to receive training from higher-level hospitals in the HVC [33].^.^

The high scores for Comprehensiveness may be due to the provision of various primary care services included in the public health service package that was implemented after China’s 2009 health care system reform. The policy requirements of equalized basic public health services meant that grassroots medical institutions had to offer a wrapped service set for residents [26]. Since village doctors in HVCs offer the public service package under the supervision of township hospitals, and their salaries and incentives depend on their performance as assessed by township hospitals, they are motivated to actively provide comprehensive public health services. In contrast, local governments purchase public health services from PVCs and VVCs, and subsidize them in accordance with the performance assessment. The subsidies and reimbursements from government are not enough to compensate for the provision of public health services in PVCs and VVCs, and additionally, the doctors in PVCs and VVCs receive less supervision and stewardship from the township hospitals compared to the doctors in HVCs [34,35]. Insufficient government subsidy and limited supervision and stewardship from superior hospitals engenders poorer comprehensive service provision in PVCs and VVCs. This then implies that providing better comprehensive service provision requires government subsidies and township hospital’s supervision for PVCs and VVCs [34–37]. Further studies are needed to identify a reasonable amount of compensation and to develop an effective performance assessment mechanism to motivate the township hospitals to grant timely and fair subsidies, and to encourage PVCs and VVCs to actively provide public health services. It is common for township hospitals to invest money in the information system construction at the affiliated HVC, thus HVCs may have received higher scores from their information systems.

The findings that PVCs performed the best on Accessibility and Continuity are perhaps due to these clinics having sole responsibility for profits and losses, and thus motivated to provide more medical services to earn more profits. Most village doctors in PVCs are local residents, so mutually connected kindred relationship, tight neighborhood relationship, and mutual trust generated from rural culture, contributed to a very close patient-physician relationship. The village doctors almost have no working time limits and are always ready to attend to a patient’s call, which makes their service extremely accessible and convenient. In contrast, the income of a HVC is handed over to the township hospitals, so these doctors have little incentive to provide extra medical services. Performance-based incentive plans should be developed to provide stronger motivation for village doctors to provide accessible services. Furthermore, some doctors at the HVCs are sent by hospitals and are not local residents, thus they have regular working hours and have no after-hours care. This situation would likely have influences on accessibility [37–38]. Previous studies also proved that generalists in private clinics work longer hours and provide more services than those who work for the government, although they provide less preventive public health services ^[^^24^^,^ ^39^^]^. Similar to the PVCs, VVCs are responsible for their own profit and loss under the surveillance of a village Committee; although this kind of supervision is not as strict as the township hospitals’ oversight of HVCs. The performance of VVCs on the domain of First Contact-accessibility and Continuity fell between the HVCs and PVCs.

In terms of satisfaction, patient satisfaction with HVCs and VVCs was higher than with PVCs. With regard to the PCAT total scores and satisfaction, patients who were more satisfied with VCs also gave higher scores for the quality of the primary care services, as higher patient satisfaction was associated with higher PCAT total scores. However, their influencing factors were different, and sometimes contradictory. This may be due to the fact that PCAT is designed to measure patient’s experience of continuity, accessibility, comprehensiveness, and coordination, while satisfaction measures the extent that primary care satisfies patients’ needs. In other words, the PCAT emphasizes the evaluation of the process, whereas satisfaction focuses on the outcomes (results) [40]. The utilization of the two indicators gives a more comprehensive evaluation of the quality of primary care. Further analysis is needed to explore the relationship and mechanisms between satisfaction and the quality of basic medical care.

Villagers who frequently visited VCs and those with medical expenditures over 20% of their total household expenditures tended to perceive better primary care. Previous research showed that the more times a patient visits a basic-level health organization, the higher PCAT score that he/she would report [41]. This may be due to the fact that chronic condition management and health care for senior citizen are included in the basic public health service package, and the policy of implementing equalized public health services allowed these groups access to better primary care.

This study has several limitations. First, this research examined the quality of primary care services from the perspective of participants but neglected factors on the provider side. Future studies should assess the influence of providers and patients simultaneously. Second, the study is short on objective indicators regarding the VC’s capacity to diagnose and treat common diseases. Third, as we had no access to the exact sampling frame of patients from the sampled villages, we did not use random sampling methods to select the patients [40]. The potential selection bias may be introduced by non-randomized sampling. Fourth, this cross-sectional survey is unable to explore causality from these findings. Future studies could use a longitudinal approach to examine the causality. Lastly, this study was based on participants’ self-reporting, so recall bias might have been introduced.

## Conclusions

This study reveals that patients at HVCs perceived better primary care than patients at PVCs and VVCs. The HVCs were given higher PCAT total scores than PVCs and VVCs, especially on Coordination and Comprehensiveness, whereas PVCs performed the best on Accessibility and Continuity. The performance of VVCs on the domains of First Contact-access and Continuity fell between that of HVCs and PVCs. Patients' satisfaction with HVCs and VVCs was higher than that of PVCs. The health care reform that integrated rural grassroots medical facilities was effective in helping VCs provide better primary care services, however, more efforts are needed to increase government subsidies and improve the supervisory relationship of township hospitals over PVCs and VVCs. Performance-based incentive plans should be developed to better motivate village doctors at HVCs to provide accessible services.

## References

[1] Starfield B, Shi L. Commentary: primary care and health outcomes: a health services research challenge. Health Serv Res. 2007;42(6 Pt 1):2252–6; discussion 94–323. 17995564 10.1111/j.1475-6773.2007.00739.x PMC2151396

[2] Hung LM, Rane S, Tsai J, Shi L. Advancing primary care to promote equitable health: implications for China. International journal for equity in health. 2012;11:2 10.1186/1475-9276-11-2 22264309 PMC3282631

[3] Chen Z. Launch of the health-care reform plan in China. Lancet. 2009;373(9672):1322–4. 10.1016/S0140-6736(09)60753-4 19376436

[4] National Health and Family Planning Commission of the People's Republic of China: Administrative Regulation to Village Clinics. Available: http://www.nhfpc.gov.cn/jws/s3581/201406/55de13fd597a4918bfc42e3a5b7ff2b8.shtml. Accessed 27 June 2014.

[5] National Health and Family Planning Commission of the People's Republic of China: Suggestions on Consolidation of Essential Drug System and Administration of Basic Medical Facilities. Available: http://www.nhfpc.gov.cn/tigs/s9661/201303/d7189887321c4b79af7767b5894bbbe6.shtml. Accessed 20 Feb 2013.

[6] National Health and Family Planning Commission of the People's Republic of China China Statistical Yearbook of Health and Family Planning(2014). Peking: Peking Union Medical College Press 2014.

[7] Wang Y, Wang X, Peng Y. Influence of "county adminstration and village manangement"political scheme on the development of village clinics. Chinese Primary Health Care. 2013;27(11):25–7.

[8] National Health and Family Planning commission of the People's Republic of China: Suggesion on further promote the integration of health services in rural area. Availiable: http://www.nhfpc.gov.cn/jws/s3581/201203/fa0953e2d8324730bbaf21bca4c0534e.shtml. Accessed 7 Apr 2010.

[9] World Health Organization The world health report 2008: primary health care now more than ever. Geneva, Switzerland: World Health Organization Press: 2008.

[10] Institute of Medicine Committee on the Future of Primary C. In: Donaldson MS, Yordy KD, Lohr KN, Vanselow NA, editors. Primary Care: America's Health in a New Era. Washington (DC): National Academies Press (US),Copyright 1996 by the National Academy of Sciences.; 1996. 25121221

[11] Starfield B, Shi L, Macinko J. Contribution of primary care to health systems and health. Milbank Q. 2005;83(3):457–502. 10.1111/j.1468-0009.2005.00409.x 16202000 PMC2690145

[12] Rao M, Clarke A, Sanderson C, Hammersley R. Patients' own assessments of quality of primary care compared with objective records based measures of technical quality of care: cross sectional study. Bmj. 2006;333(7557):19 10.1136/bmj.38874.499167.7C 16793783 PMC1488754

[13] Grol R, Wensing M, Mainz J, Jung HP, Ferreira P, Hearnshaw H, et al Patients in Europe evaluate general practice care: an international comparison. Br J Gen Pract. 2000;50(460):882–7. 11141874 PMC1313852

[14] Fracolli LA, Gomes MF, Nabao FR, Santos MS, Cappellini VK, de Almeida AC. Primary health care assessment tools: a literature review and metasynthesis. Ciencia & saude coletiva. 2014;19(12):4851–60. 25388193 10.1590/1413-812320141912.00572014

[15] Malouin RA, Starfield B, Sepulveda MJ. Evaluating the tools used to assess the medical home. Managed care (Langhorne, Pa). 2009;18(6):44–8. 19569570

[16] Haggerty JL, Burge F, Beaulieu MD, Pineault R, Beaulieu C, Levesque JF, et al Validation of instruments to evaluate primary healthcare from the patient perspective: overview of the method. Healthc Policy. 2011;7(Spec Issue):31–46. 23205034 PMC3399433

[17] Shi L, Starfield B, Xu J, Politzer R, Regan J. Primary care quality: community health center and health maintenance organization. Southern medical journal. 2003;96(8):787–95. 10.1097/01.SMJ.0000066811.53167.2E 14515920

[18] Wang HH, Wong SY, Wong MC, Wei XL, Wang JJ, Li DK, et al Patients' experiences in different models of community health centers in southern China. Ann Fam Med. 2013;11(6):517–26. 10.1370/afm.1545 24218375 PMC3823722

[19] McCollum R, Chen L, ChenXiang T, Liu X, Starfield B, Jinhuan Z, et al Experiences with primary healthcare in Fuzhou, urban China, in the context of health sector reform: a mixed methods study. Int J Health Plann Manage. 2014;29(2):e107–26. 10.1002/hpm.2165 23576191

[20] Muggah E, Hogg W, Dahrouge S, Russell G, Kristjansson E, Muldoon L, et al Patient-reported access to primary care in Ontario: effect of organizational characteristics. Canadian family physician Medecin de famille canadien. 2014;60(1):e24–31. 24452575 PMC3994832

[21] Tsai J, Shi L, Yu WL, Lebrun LA. Usual source of care and the quality of medical care experiences: a cross-sectional survey of patients from a Taiwanese community. Medical care. 2010;48(7):628–34. 10.1097/MLR.0b013e3181dbdf76 20548255

[22] Wei X, Li H, Yang N, Wong SY, Owolabi O, Xu J, et al Comparing quality of public primary care between Hong Kong and Shanghai using validated patient assessment tools. PloS one. 2015;10(3):e0121269 10.1371/journal.pone.0121269 25826616 PMC4380428

[23] Russell G, Dahrouge S, Tuna M, Hogg W, Geneau R, Gebremichael G. Getting it all done. Organizational factors linked with comprehensive primary care. Fam Pract. 2010;27(5):535–41. 10.1093/fampra/cmq037 20534791

[24] Wei X, Yang N, Gao Y, Wong SY, Wong MC, Wang J, et al Comparison of three models of ownership of community health centres in China: a qualitative study. J Health Serv Res Policy. 2015;20(3):162–9. 10.1177/1355819615579700 25899485

[25] Shi L, Starfield B, Xu JH. Validating the adult primary care assessment tool. Journal of Family Practice. 2001;50(2):161–74w.

[26] National Health and Family Planning commission of the People's Republic of China: Suggesion on promote equal access to basic public health services for all. Availiable: http://www.nhfpc.gov.cn/jws/s3577/200907/41745.shtml Accessed 14 Jul 2009.

[27] Liu X, Qiao Y, Li q. Research on major organizers of village clinics. Chinese Primary Health Care. 2014;28(11):1–3.

[28] Meng Q, Liu X, Shi J. Comparing the services and quality of private and public clinics in rural China. Health Policy Plan. 2000;15(4):349–56. 11124237 10.1093/heapol/15.4.349

[29] Sung NJ, Suh SY, Lee DW, Ahn HY, Choi YJ, Lee JH. Patient's assessment of primary care of medical institutions in South Korea by structural type. Int J Qual Health Care. 2010;22(6):493–9. 10.1093/intqhc/mzq053 20935007

[30] Wong SY, Kung K, Griffiths SM, Carthy T, Wong MC, Lo SV, et al Comparison of primary care experiences among adults in general outpatient clinics and private general practice clinics in Hong Kong. BMC Public Health. 2010;10:397 10.1186/1471-2458-10-397 20602806 PMC2908092

[31] Yang H, Shi L, Lebrun LA, Zhou X, Liu J, Wang H. Development of the Chinese primary care assessment tool: data quality and measurement properties. Int J Qual Health Care. 2013;25(1):92–105. 10.1093/intqhc/mzs072 23175535

[32] Starfield B, Shi L. Manaual for the primary care assessment tools. Baltimore: John Hopkins University Press 2009.

[33] Y X, Z F, M T, X Z. Research on the effect assessment of township and village health services integration policy in the minority-inhabited aresa in western China. International Journal of Integrated Care. 2014;14(9):182–4.

[34] Ding Y, Smith HJ, Fei Y, Xu B, Nie S, Yan W, et al Factors influencing the provision of public health services by village doctors in Hubei and Jiangxi provinces, China. Bull World Health Organ. 2013;91(1):64–9. 10.2471/BLT.12.109447 23397352 PMC3537250

[35] Yip W, Hanson K. Purchasing health care in China: experiences, opportunities and challenges. Advances in health economics and health services research. 2009;21:197–218. 19791704

[36] Zhang S, Zhang W, Zhou H, Xu H, Qu Z, Guo M, et al How China's new health reform influences village doctors' income structure: evidence from a qualitative study in six counties in China. Human resources for health. 2015;13:26 10.1186/s12960-015-0019-1 25940189 PMC4440293

[37] Zhang Y, Chen M, Shi XQ. Challenges of basic public health services provided by village doctors in Guizhou, China. Asia-Pacific journal of public health / Asia-Pacific Academic Consortium for Public Health. 2015;27(2 Suppl):69s–76s. 10.1177/1010539514568708 25673280

[38] Li s. Rearch on the relationship between village cultural and the rural primary health. Peking: Publishing House Press; 2014.

[39] Richard BS, Ana R, Wienke B. Primary care in the driver's seat? Organizational reform in European primary care Peking: McGraw-Hill Education(Asia) and China Labour and Social Security Publishing House; 2010.

[40] Li H, Wei X, Wong MC, Wong SY, Yang N, Griffiths SM. A Cross-Sectional Comparison of Perceived Quality of Primary Care by Hypertensive Patients in Shanghai and Shenzhen, China. Medicine. 2015;94(34):e1388 10.1097/MD.0000000000001388 26313780 PMC4602902

[41] Wei X, Li H, Yang N, Wong SY, Chong MC, Shi L, et al Changes in the perceived quality of primary care in Shanghai and Shenzhen, China: a difference-in-difference analysis. Bull World Health Organ. 2015;93(6):407–16. 10.2471/BLT.14.139527 26240462 PMC4450701

